# Novel drug-regulated transcriptional networks in brain reveal pharmacological properties of psychotropic drugs

**DOI:** 10.1186/1471-2164-14-606

**Published:** 2013-09-08

**Authors:** Michal Korostynski, Marcin Piechota, Jaroslaw Dzbek, Wiktor Mlynarski, Klaudia Szklarczyk, Barbara Ziolkowska, Ryszard Przewlocki

**Affiliations:** 1 Department of Molecular Neuropharmacology, Institute of Pharmacology Polish Academy of Sciences, Smętna 12, PL 31-343, Kraków, Poland

**Keywords:** Gene expression patterns, Psychotropic drugs, Striatum, Molecular classification

## Abstract

**Background:**

Despite their widespread use, the biological mechanisms underlying the efficacy of psychotropic drugs are still incompletely known; improved understanding of these is essential for development of novel more effective drugs and rational design of therapy. Given the large number of psychotropic drugs available and their differential pharmacological effects, it would be important to establish specific predictors of response to various classes of drugs.

**Results:**

To identify the molecular mechanisms that may initiate therapeutic effects, whole-genome expression profiling (using 324 Illumina Mouse WG-6 microarrays) of drug-induced alterations in the mouse brain was undertaken, with a focus on the time-course (1, 2, 4 and 8 h) of gene expression changes produced by eighteen major psychotropic drugs: antidepressants, antipsychotics, anxiolytics, psychostimulants and opioids. The resulting database is freely accessible at http://www.genes2mind.org. Bioinformatics approaches led to the identification of three main drug-responsive genomic networks and indicated neurobiological pathways that mediate the alterations in transcription. Each tested psychotropic drug was characterized by a unique gene network expression profile related to its neuropharmacological properties. Functional links that connect expression of the networks to the development of neuronal adaptations (MAPK signaling pathway), control of brain metabolism (adipocytokine pathway), and organization of cell projections (mTOR pathway) were found.

**Conclusions:**

The comparison of gene expression alterations between various drugs opened a new means to classify the different psychoactive compounds and to predict their cellular targets; this is well exemplified in the case of tianeptine, an antidepressant with unknown mechanisms of action. This work represents the first proof-of-concept study of a molecular classification of psychoactive drugs.

## Background

The complex etiology and heterogeneity of mental disorders is associated with moderate effectiveness of psychoactive drugs, frequent recurrence of symptoms and high cost of therapy [1]. Psychotropic drugs have diverse therapeutic profiles (Table 1), and even a single class drugs can show high diversity of effectiveness and effects may be limited to particular sub-types of a given disorder, exemplified by the various subclasses of antidepressants [2]. On the other hand, drugs belonging to different therapeutic classes may have effects that are either beneficial or adverse in a particular disease. Therefore, the identification of common and specific neurobiological actions of psychoactive compounds is critical to understanding therapeutic mechanisms. Furthermore, comparison of drug-induced molecular profiles may provide objective criteria for a more rational classification of psychotropic drugs.

**Table 1:** A list of psychotropic drugs selected for the comparison

	**Drug**	**Dose [mg/kg, i.p.] (control)**^**1**^	**Pharmacological targets**	**Clinical group**^**7**^
1	Mianserin (MIA)^3^	20 (sal)	HRH1 / HTR2C, 2A, 3A / ADRA2C, 2A, 1A / NET / CHRM	Antidepressant (NaSSa)
2	Imipramine (IMI)^3^	10 (sal)	SERT / HRH1 / NET / ADRA1A / HTR2C / CHRM	Antidepressant (TCA)
3	Fluoxetine (FLU)^3^	20 (sal)	SERT / NET / HTR2C	Antidepressant (SSRI)
4	Bupropion (BUP)^3^	20 (sal)	DAT	Antidepressant (DRI)
5	Tianeptine (TIA)^2^	20 (sal)	*Unknown targets*	Antidepressant (SSRE)
6	Tranylcypromine (TRA)^2^	20 (sal)	MAO	Antidepressant (MAOI)
7	Methamphetamine (MET)^2^	2 (sal)	NET / DAT	Psychostimulant
8	Cocaine (COC)^2^	25 (sal)	DAT / NET / SERT	Psychostimulant
9	Nicotine (NIC)^2^	1 (sal)	n-AChR	Psychostimulant
10	Heroin (HER)^6^	10 (sal)	OPRM1, OPRK1, OPRD1	Analgesic
11	Morphine (MOR)^4^	20 (sal)	OPRM1, OPRK1, OPRD1	Analgesic
12	Ethanol (ETO)^2^	2000 (sal)	GABAA	Anxiolytic/Analgesic
13	Diazepam (DIA)^2^	5 (twe)	GABAA	Anxiolytic
14	Buspirone (BUS)^3^	10 (sal)	5HT1A	Anxiolytic
15	Hydroxizine (HYD)^2^	10 (sal)	HRH1	Anxiolytic
16	Clozapine (CLO)^3^	3 (twe)	DRD4, 2 / HRH1 / HTR2A, 2C / DRD1 / CHRM / ADRA1A	Antipsychotic
17	Risperidone (RIS)^3^	0.5 (twe)	HTR2A, 2C / DRD1 / DRD2, 3, 4 / ADRA1A, 2C / HRH1	Antipsychotic
18	Haloperidol (HAL)^3^	1 (twe)	DRD2, 3, 4 / 5HT2	Antipsychotic
19	Tween 80^2^	vol. 10 ml/kg (naïve)	*No targets*	Vehicle
20	Saline (SAL)^5^	vol. 10 ml/kg (naïve)	*No targets*	Vehicle

^1^the effective drug doses were based on the literature; the drugs were purchased from ^2^Sigma-Aldrich, ^3^Biotrend, ^4^Teva, ^5^Polpharma or ^6^synthesized from morphine, ^7^class of antidepressant (*NaSSa*, noradrenergic and specific serotonergic antidepressant, *TCA* tricyclic antidepressant, *SSRI* selective serotonin reuptake inhibitor, *DRI* dopamine reuptake inhibitor, *SSRE* selective serotonin reuptake enhancer, *MAOI* monoamine oxidase inhibitor).

The development of maladaptive neuroplastic changes is suggested to underlie the progression of neuropsychiatric disorders [3]. The pattern of structural alterations in the brain is determined by the process of synaptic plasticity and is influenced by genetic, neurodevelopmental and environmental factors [4]. It is thought that therapeutic agents reverse disease-related alterations by reconstruction and normalization of neuronal connections in targeted brain areas [5,6]. This view is supported by the fact that the therapeutic effects of psychotropic drugs usually have delayed onset and gradually increase with time. Establishment of these long-lasting changes requires gene expression and synthesis of new proteins [7-9] in a time-dependent and region-specific manner; such changes may serve as early markers of drug-activated biological processes.

Impaired control over drive and motivation is a frequent symptom in psychiatric disorders, including depression, mania and addiction [10,11]. Since these conditions are characterized by disturbed monoaminergic function, most current pharmacotherapies target receptors and transporters for dopamine, serotonin and noradrenaline as well as other transmitters such as GABA and acetylcholine [12,13]. All of these transmitter systems are represented in the striatum, a brain region responsible for control of motivation, reward-based learning and decision-making [14,15]. The striatum as an evolutionarily ancient brain region reveals comparable functions and gene expression profiles between rodents and humans [16]. Thus, despite the limitations of an animal model [17], the comparison of drug-induced dynamic alterations in the rodent striatal gene expression profile provides insights into molecular mechanisms of psychotropic drug actions.

In this study, using whole-genome gene expression microarrays we identified main drug-responsive genomic networks that are regulated by 18 individual psychoactive drugs known to impact on one or more pharmacological targets within the striatum. This work introduces a novel approach for the classification of psychotropic drugs on the basis of gene expression profiling. To encourage further discoveries along these lines, we made freely available the entire interactive database which contains the results of the present study (http://www.genes2mind.org).

## Results

### Drug-induced transcriptional alterations in the striatum

Using whole-genome microarrays (Illumina MouseWG-6), we compared striatal gene expression profiles (magnitude and dynamics of ~30,000 genes) produced by 18 major psychoactive drugs at 1, 2, 4 and 8 hours after acute administration (Table 1). Obtained data were subjected to two-way analysis of variance (ANOVA) with *drug* and *time* as factors (Additional file 1). We found 317 drug-responsive transcripts in the striatum at the most conservative statistical threshold (P < 0.05 after Bonferroni correction, nominal P = 1.36 × 10^-06^). After removal of genes represented more than once on the microarrays, this list contains 278 unique genes. For down-stream analyses we selected top 300 transcripts ordered by genes2mind score, which takes into account fold of change and direction of drug-induce gene expression alterations (please see Methods for details). This method was implemented in the genes2mind selection module (http://www.genes2mind.org). Furthermore, we estimated total number of genes regulated by psychotropic drugs by calculation of true positives over a wide range of false discovery rates (FDR). This estimation indicated that the total number of regulated transcripts slightly exceeds one thousand (Additional file 2). Therefore, with the restrictive statistical approach we identified about 30% of drug-responsive genes in the striatum. For canonical pathway analysis we used 5% FDR threshold at which we identified most of drug-regulated transcripts (872 microarray probes). The number of genes regulated by each drugs in the time-course (genes2mind score > 10) is presented in Additional file 3. All the additional analyses and comparisons (including selection of drugs, genes and time-points) are available at the genes2mind resource.

### Molecular classification of psychotropic drugs

**Figure 1:**
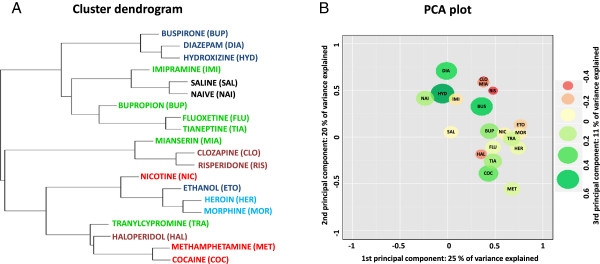
**A comparison of psychotropic drugs based on pattern of gene expression alterations in the striatum.** Cluster dendrogram **(A)** and PCA plot **(B)** were generated based on expression profile of top 300 drug-responsive genes. Distance between drugs corresponds to a divergence in the profile of drug-induced transcriptional alterations. Therapeutic classes of drugs are coded by colors presented on the clustering (green for antidepressants, red for psychostimulants, blue for anxiolytics, brown for antipsychotics and light blue for opioids). First and second components of PCA are shown on x and y axis, while third component is coded by color and size of the circles as presented on the right.

We used hierarchical clustering and principal component analysis (PCA) of the 300 drug-responsive transcripts (defined by genes2mind score using all the time-points) to classify psychotropic drugs. Drug-induced transcriptional signatures were distinguished between the various therapeutic groups: anxiolytics (buspirone, diazepam and hydroxyzine), atypical antipsychotics (clozapine and risperidone), opioids (morphine and heroin) and psychostimulants (methamphetamine and cocaine) (Figure 1A). However, the expression profile of the antipsychotic drug - haloperidol was similar to that of psychostimulants and tranylcypromine. Also, the effects of nicotine resembled those of addictive drugs, ethanol and opioids, more closely than other psychostimulants. Antidepressants proved to be the most heterogeneous group of drugs in terms of their impact on gene expression, with mianserin, imipramine, tranylcypromine and fluoxetine displaying very diverse profiles. The gene expression profile of mianserin was most similar to those elicited by atypical neuroleptics; the profiles obtained in response to imipramine were similar to those produced by anxiolytics; and tranylcypromine generated a profile that resembled that obtained with psychostimulants. Nevertheless, antidepressants that target monoamine transporters (fluoxetine and bupropion) fell into one cluster.

Three main PCA components explained 56% of the variance in gene expression and mapped the test drugs in three-dimensional space according to their molecular profiles (Figure 1B). The first PCA component represented the strong effects of opioids, ethanol and tranylcypromine; there were no detectable effects of diazepam and hydroxyzine. The second principal component included the full spectrum of drug-induced effects in the striatum - from substantial inhibition of gene expression by diazepam or clozapine to strong activation by cocaine and methamphetamine. The third PCA component showed, on one hand, a common effect of antipsychotic drugs and mianserin, and on the other, a common effect of all three anxiolytics.

### Drug-induced gene expression networks

**Figure 2:**
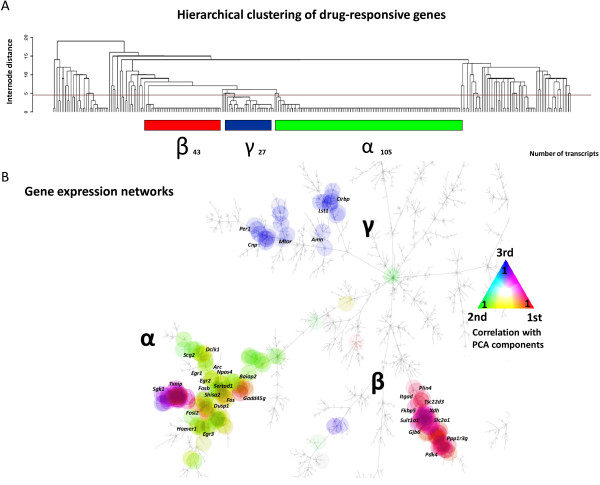
**Drug-induced gene expression networks in the striatum.** Gene networks α, β, γ were identified by hierarchical clustering of top drug-regulated transcripts **(A)**. Map of the striatal transcriptome represented as minimal spanning tree **(B)**. Each node represents one transcript. The inter-node distance is proportional to the Spearman correlation of the expression levels of two genes. The node color of drug-responsive transcripts indicates the level of correlation between gene expression and PCA components (see Figure 1b), according to a triangle scale.

**Figure 3:**
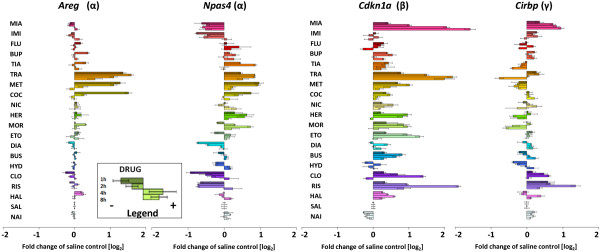
**Psychotropic drug-induced transcriptional alterations of example genes.** Four genes with typical expression profiles for the networks α (*Areg* and *Npas4*), β (*Cdkn1a*) and γ (*Cirbp*) were selected from drug-responsive transcripts. The microarray results are presented as time-course (1, 2, 4 and 8 hours) of fold-changes vs. saline control (as described in the legend). Results from drug-naïve group of animals are presented as an additional control.

To connect effects of psychotropic drugs to inducible gene expression patterns in the striatum, we determined the correlation between loadings of the first three PCA components and the level of transcriptional alterations. Hierarchical clustering was used to search for drug-inducible groups of co-expressed genes (Figure 2A). Three main drug-responsive gene clusters, representing network α (containing 105 transcripts), β (43 transcripts) and γ (27 transcripts) became evident. The clusters revealed diverse drug- and time-dependent patterns of up- and down-regulation of gene expression (see Figure 3 for examples of typical genes). Next, a map of the complete striatal transcriptome, based on the level of correlation between profiles of all transcripts measured using microarrays, was developed in order to depict drug-induced alterations in expression. All three drug-responsive gene clusters are located on the same branch of a tree (Additional file 4). The clusters were clearly separated and organized in drug-regulated genomic networks (Figure 2B). We found no other networks with distinct gene expression patterns. However, it is possible to identify subclusters of genes with moderately different profiles of expression within the three main networks (Additional file 5).

Expression of gene network α correlated with the second PCA component. Drug-induced changes in network α genes were bidirectional, e.g. methamphetamine induced expression of *Npas4* and *Egr1*, while diazepam inhibited it. Buspirone, mianserin and risperidone induced some activity-dependent genes but down-regulated others, e.g. mianserin inhibited expression of *Homer1* by 0.6-fold and induced *Fos* by 2.3-fold. The gene expression network β correlated with the first PCA factor. The expression of this network was regulated to a different degree by drugs from various pharmacological groups, e.g. *Cdkn1a* or *Fkbp5* after opioids and tranylcypromine, except that diazepam, hydroxyzine and imipramine had no effects. The expression of gene network γ correlated with the third PCA component. All network γ genes were regulated by risperidone, mianserin and clozapine, in a bidirectional manner, e.g. *Cirbp* and *Mtor* were strongly up-regulated by atypical neuroleptics and mianserin, but down-regulated by tranylcypromine or methamphetamine.

### Functional description of drug-regulated gene networks

#### Cell-type enrichment of drug-responsive genes

**Figure 4:**
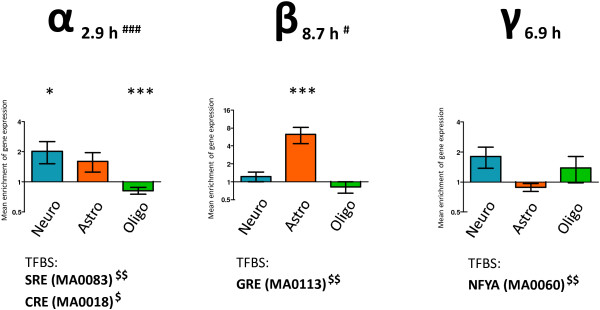
**Functional analysis of drug-induced gene networks α, β and γ.** The networks were described by gene enrichment in neurons, astrocytes and oligodendrocytes, overrepresentation of transcription factor binding sites (TFBS) and median mRNA half-life (median for whole-transcriptome = 5.6 h); significance of enrichment: *P < 0.05, **P < 0.01, ***P < 0.001.

Identification of the types of neural cells expressing genes from the α, β and γ networks was carried out by reference to publicly available data that represents cellular enrichment of individual transcripts in neurons, astrocytes or oligodendrocytes (Figure 4) [18]. Significant enrichment of transcripts from the expression network α was found in neurons (e.g. neuron-specific *Npas4*, *Scg2* or *Baiap2*) and under-representation in oligodendrocytes. Gene network β was characterized by very strong over-representation of genes expressed in astrocytes, including the cell-type specific markers *Gjb6* and *Ppp1r3g*. Network γ genes did not show enrichment in any particular cell type although individual genes that were highly expressed in either neurons (*Amn* and *Lst1*) or oligodendrocytes (*Cnp*) were identified.

#### Over-representation of transcription factor binding sites

The cREMaG database [19] was used for *in silico* identification of molecular factors involved in the transcriptional control of the gene expression networks revealed in the present study. We found significant over-representation of SRE (serum-response elements, 4-fold enrichment; P < 0.01) and CRE (cAMP response elements, 3.8-fold enrichment, P < 0.05) in the promoter regions of genes from network α. These elements are potential binding sites for the transcriptional factors SRF (e.g. in *Egr1* and *Arc*) and CREB1 (e.g. in *Dusp1* and *Fosl2*). Significant over-representation of GRE (glucocorticoid-response elements, 4.3-fold enrichment, P < 0.01) on promoter regions of genes from network β (e.g. in *Tsc22d3* and *Pdk4*) was observed. Gene network γ showed significant enrichment of binding sites for transcriptional factor NFYA (5.8-fold enrichment, P < 0.01). Two examples of genes with conserved binding sites for NFYA (regulatory subunit for NF-Y complex) are *Per1* and *Mtor*.

#### Transcript stability of drug-responsive genes

Transcript stability is related to function of the transcribed protein [20]. Our analysis reveals substantial differences in the half-lives of mRNAs belonging to the α and β networks. Gene expression network α contains genes with a short mRNA half-life (median = 2.9 h), including very short-lived transcripts (< 2 h half-life: *Gadd45g*, *Fos* and *Egr2*). In contrast, network β includes transcripts with significantly longer (median: 8.7 h) half-lives (> 20 h in the case of *Sult1a1* and *Itgad*). Whole-genome screening indicated that genes with low mRNA stability are frequently involved in regulation of intracellular signaling, while long-lived transcripts have a role in cell metabolism [20]. The median half-life of transcripts from network γ was 6.9 h, i.e. not significantly different from the median of 5.6 h for the whole transcriptome.

#### Functional classification of drug-responsive genes

To characterize the transcriptional representation of biological processes, a list of genes from each gene expression network was analyzed using GO (Gene Ontology). Functional clusters of transcripts connected with protein MAP kinase phosphatase activity (81-fold enrichment, P = 2.4 × 10^-9^; e.g., *Dusp1* and *Dusp4*), rhythmic processes (7.2-fold, P = 2.3 × 10^-3^; e.g., *Egr2* and *Per2*) and transcriptional regulator activity (2.8-fold, P = 6.7 × 10^-5^; e.g., *Sertad1* and *Atf3*) were over-represented among genes from network α. The group of genes from network β was enriched in transcripts involved in lipid metabolism (11.5-fold, P = 2.2 × 10^-3^; e.g., *Adipor2* and *Pnpla2*) and formation of adherens junctions (13.6-fold, P = 1.3 × 10^-3^; e.g., *Dlg5* and *Synm*), whereas, analysis of the novel network γ revealed the enrichment of genes connected to cell projection organization (4.7-fold, P = 8.5 × 10^-3^; e.g., *Lst1* and *Cnp*). A detailed description of the results of GO classification is included in Additional file 6.

We did not find transcriptional regulation of genes coding main targets for psychotropic drugs, as for example dopamine receptors *Drd2* and *Drd1a* or serotonin transporter *Slc6a4*. It is possible that promoters of these genes are not directly activated in response to the ligand binding.

#### Canonical pathways analysis

A canonical pathways analysis was performed to investigate the functional characteristics between drug-regulated genes. To increase resolution of the analysis an extended list of transcripts was used (872 transcripts at <5% FDR). Genes were assigned to the networks according to drug-induced profiles of alterations in expression (Additional files 1 and 5). The canonical pathways analysis using the Pathways-Express identified significant biological functions altered differentially by the psychotropic drugs at the statistical threshold of P < 0.05. The Additional file 7 contains a list of biological pathways for each drug-regulated transcriptional network. The list of pathways for gene expression network α includes neuroplasticity-related signaling cascades MAPK and ErbB. The network β contains genes involved in the control of cellular metabolism via glucose regulation by adipocytokine and PPARG molecular pathways. The γ network proved to be enriched in genes involved in regulation of circadian rhythm and mTOR signaling pathways. Additional file 8: Figure S8 shows examples of canonical pathways enriched between drug-responsive genes (Additional file 8).

### The examples of drug-regulated transcriptional profiles

We selected genes with expression patterns representative for the identified drug-responsive transcriptional networks (Figure 3). In general, transcripts from network α reveal remarkable correlation in expression profile, but we also found drug-dependent diversity between particular genes.

The expression of *Areg* is induced by psychostimulants and tranylcypromine administration. The profile of *Areg* expression suggests that an increase in dopaminergic transmission may be directly involved in drug-induced regulation of this gene. Moreover, the time-course of alterations in *Areg* expression corresponds to pharmacokinetic properties of the drugs: we observed a strong increase in the mRNA abundance of *Areg* 1 h after injection of cocaine, 2 h after methamphetamine and 4 h after tranylcypromine. *Npas4* is induced by these three drugs in the same direction as *Areg*, but unlike *Areg*, *Npas4* expression is also induced by opioids and inhibited by diazepam, atypical neuroleptics as well as antidepressants that antagonize 5-HT2 receptors. Other examples of genes with slightly different profiles are *Egr2* and Arc, with expression induced by haloperidol and buspirone as well as *Dusp1* and *Fos*, induced by haloperidol and mianserin (http://www.genes2mind.org).

Drug-induced network β reveals more homogenous pattern of transcriptional alterations. Genes from this network showed the largest alterations (up to 6-fold vs. saline control group) in expression 4 h after administration of mianserin and tranylcypromine. Example gene *Cdkn1a* is activated to a different degree by all the psychotropic drugs, except for imipramine, diazepam and hydroxyzine.

Network γ exhibits a pattern of gene expression changes that is connected to pharmacological properties of the drugs. Transcriptional activation of *Cirbp* is limited to mianserin, risperidone and clozapine treatment while opioids and psychostimulants seem to inhibit the expression of this gene and other network γ genes (e.g., *Trove2* and *Mrpl15*).

### Prediction of drug-target interactions from gene expression profiling

**Figure 5:**
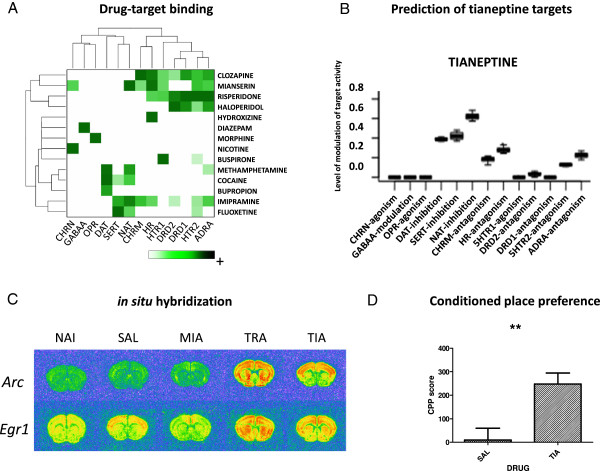
**Prediction of tianeptine targets by gene expression profiling.** A matrix of drug-target binding interactions from the PDSP Ki database **(A)**. The mechanisms of tianeptine action predicted from expression profiles of the transcripts most sensitive to the analyzed pharmacological mechanisms **(B)**. Brain distribution of tianeptine-induced gene expression alterations of *Arc* and *Egr1* and **(C)**. Rewarding properties of tianeptine in the conditioned place preference test. Scores are expressed as means + standard error of the mean (t-test, **P < 0.01) **(D)**.

The comparison of gene expression profiles has been recently used as a tool for prediction of therapeutic properties of drugs [21-23]. Our molecular classification of psychotropic drugs indicated an interesting profile of tianeptine. Tianeptine is a tricyclic antidepressant whose mechanism of action is still not clear [24]. Here, a linear model, based on the level of gene expression alterations induced in the striatum by drugs with well-known pharmacological properties (Figure 5A), was used in an attempt to predict tianeptine’s molecular mechanism(s) of action. The matrix of interactions between 14 psychotropic drugs and 13 neuropharmacological mechanisms was constructed on the basis of data in the PDSP Ki database [25]. Levels of modulation by tianeptine were predicted for each of the mechanisms. This analysis revealed that the transcriptional effects of tianeptine may involve increased activity of noradrenaline, serotonin and dopamine neurotransmission (Figure 5B).

### Monoaminergic action of tianeptine

Further, *in situ* hybridization was used to examine the anatomical distribution of drug-induced alterations in the expression of two neuroplasticity-related genes following exposure of mice to tianeptine and two other antidepressants, tranylcypromine and mianserin. Patterns of tianeptine-induced expression of *Arc* and *Egr1* in the forebrain proved similar to those produced by tranylcypromine, an inhibitor of monoamine oxidase that increases the concentrations of all monoamine neurotransmitters; both drugs induced *Arc* and *Egr1* transcription in the striatum and neocortex (Figure 5C). In contrast, mianserin, which affects levels of noradrenaline, but not of dopamine or serotonin, produced different effects; these included downregulation of *Arc* and *Egr1* transcripts in the striatum. According to these profiles, we predicted that tianeptine, like other monoamine stimulants, may have a positive reinforcing effect in animals. This prediction was confirmed in the conditioned place preference test in which we observed a significant increase in time spent in the environmental context associated with tianeptine administration (P < 0.01, t-test) (Figure 5D). Thus, both the patterns of drug-induced gene expression and the behavioral data support the conclusion that tianeptine acts as a positive modulator of monoaminergic neurotransmission.

## Discussion

The profile of drug-induced gene expression in the brain is determined by activity of different neurotransmiter systems and response of various types of cells. To unravel complexity of this profile we designed a detailed time-course gene expression study for eighteen psychotropic drugs belonging to all the major clinical classes. The previously published large-scale gene expression analyses were focused on a single drug [26,27], drugs from one clinical class [28,29] or marker genes [30,31]. Transcriptome alterations induced in the brain by buspirone, bupropione, hydroxyzine or tianeptine were not analyzed so far. The study involved extraction of a pool of approximately 1000 transcripts that are regulated by psychotropic drugs. Differential transcription of different subsets of genes from this pool was observed during the first few hours after drug administration; 90% of the affected transcripts were up-regulated and the remaining were inhibited. Interestingly, the majority of drug-induced transcriptional alterations dissipated within 8 hours of treatment, indicating that drug-induced changes of mRNA abundance are transient. We suppose that they are then rapidly followed by protein expression and these proteomic alterations translate short-lived transcriptional drug effects into lasting structural modifications of the brain; further, we suggest that chronic drug treatment leads to accumulation of drug-induced plastic alterations that eventually become manifest as therapeutic effects.

This study was limited to transcriptional mechanisms activated in response to acute drug administration. The effects of psychoactive drugs such as anti-psychotic action, mood normalization, tolerance or addiction require repeated treatment. We have recently investigated gene expression changes at several time-points after chronic administration of heroin or methamphetamine to associate drug-induced molecular changes with long-term behavioral adaptations e [32]. In that work we found that effects of chronic treatment share transcriptional alterations with single administration, as for example regulation of glucocorticoid-dependent (*Plin4* and *Fkbp5*) or circadian rhythm-regulated genes (*Per1* or *Per2*). However, there were no direct correlations between transcriptional and behavioral effects of the drugs as well as no significant changes in gene expression profile after a period of withdrawal. We conclude that psychoactive drugs induce transient transcriptional program that may initiate neuroplastic alterations, but does not trigger long-term alteration in mRNA abundance levels in mature differentiated brain cells.

Bioinformatic analysis revealed that the transcriptional response to the psychotropic drugs tested fall into three major groups of co-regulated genes. The largest gene network, α, contains genes previously defined as being activity-dependent. The observed alterations in expression of genes belonging to the network α correspond well to drug effects on neuronal activity (e.g. activation by cocaine and inhibition by diazepam); expression of genes in this network is known to depend on an interplay between the transcriptional factors CREB and SRF [33-35]. The gene network α includes a number of neuroplasticity-related transcriptional factors (e.g. *Npas4*, *Egr1* and *Fosb*) as well as other regulators of brain plasticity (e.g. *Arc* and *Homer1*) [36-39]. Moreover, some network α genes are involved in MAP kinase signal transduction pathway (e.g. *Dusp1* and *Dusp6*) which plays a pivotal role in various forms of long-lasting neuroplasticity [40,41]. The network α also contains novel genes (e.g. transcripts related to ErbB receptors signaling pathway *Ddit4*, developmental neuronal death *Sertad1* or a modulator of Wnt and Fgf signaling pathways *Shisa2*) [42,43]. These genes deserve further functional characterization with respect to drug effects. All network α genes were shown to be expressed in neurons and their mRNAs were found to have relatively short half-lives. Several lines of evidence indicate that the expression of genes belonging to network α is involved in the initiation of plastic alterations and long-term modulation of neuronal signaling and the diverse functions of these genes indicate that psychotropic drugs activate control points for multiple intracellular pathways [44]. Accordingly, we suggest that, at the transcriptional level, brain plasticity is regulated through expression of molecular switches rather than of all components of neuroplasticity-related pathways.

Another network identified (β) is strongly enriched in genes that are expressed predominantly in astrocytes and glucocorticoid response elements (GRE) in their promoter regions are overrepresented. However, while the collective function of network β genes in astrocytes remains unknown, genes from this group are implicated in glucose metabolism e.g. *Pdk4* and glucose transport e.g. *Slc2a1* as well as other metabolic processes; in addition, *Sult1a1* is involved in sulfate conjugation of neurotransmitters and certain xenobiotics and *Xdh* plays a role in the oxidative metabolism of purines [45-48]. The network β is enriched for genes related to adipocytokine signaling pathway. This molecular cascade is an important regulator of energy intake and metabolic rate [49]. It thus appears that glial cells use expression of network β genes to activate a set of metabolic control points and therefore, to support the functional responses of neurons to psychotropic drugs. The relatively long half-lives of the mRNAs generated from these genes most likely contribute to the regulation of neural cell metabolism [20]; interestingly, patients with affective disorders often display altered brain metabolism [50]. Glucocorticoids are key regulators of cellular metabolism and their dysregulated secretion is found in several psychiatric disorders [51]; in major depression, antidepressant actions are usually first seen only after glucocorticoid secretion has been normalized [52]. Thus, activation of glucocorticoid-dependent genes following psychotropic drug treatment may represent restoration of homeostatic control of brain metabolism.

The third psychotropic drug-inducible network, γ, that emerged from this study includes genes involved in the organization of cell projections (e.g. *Lst1*, *Cnp*) and the mTOR pathway (e.g. *Mtor*, *Tsc1*) [53-55]. Evolving evidence implicates the mTOR pathway in dendrite arborization and spine morphology [56]. Network γ may therefore serve to switch on multiple control points for morphological alterations in nerve cells. Our results indicate that expression of this network in the striatum may depend on serotonin signaling, specifically the 5-HT2 receptor. Thus, gene network γ may be involved in the mediation of the long-lasting effects of 5-HT2 antagonist antipsychotic drugs on the cellular level. Moreover, the 5-HT2 blockade-dependent expression of network γ in the striatum that separates haloperidol from risperidone may be useful as a transcriptional marker for atypical neuroleptics.

The current Anatomical Therapeutic Chemical (ATC) and World Health Organization (WHO) classification of psychotropic drugs is based on their clinical effectiveness. As shown by the present work, comparison of gene expression profiles can clearly distinguish between atypical antipsychotics, opioids and psychostimulants. Moreover, the three anxiolytic drugs studied here show relatively similar genomic profiles despite the different mechanisms associated with their actions. Interestingly, these anxiolytics and the antidepressant imipramine share a common expression profile and imipramine can act efficiently to reduce anxiety [57]. On the other hand, the molecular profile of mianserin differs markedly from that of imipramine while being similar to that of the atypical antipsychotics clozapine and risperidone; this may reflect the fact that all of these drugs can modulate serotonergic activity. The potential utility of the presently-described approach to distinguish between the two classes of antipsychotics is further illustrated by the finding that the typical antipsychotic haloperidol has a similar molecular profile to that of psychostimulant drugs. This most likely results from the propensity of all these drugs to upregulate activity-dependent genes in the striatum. It is important to note, however, that haloperidol and psychostimulants induce these genes in different neuronal populations and via different pharmacological mechanisms [58]. Another observation from the present analysis is that drugs which trigger large increases in striatal dopamine and norepinephrine levels induce similar expression profiles (cocaine and methamphetamine, tranylcypromine).

In general, antidepressants proved to be highly heterogeneous with respect to activation of molecular networks. This diversity reflects their diverse pharmacological and neurobiological mechanisms of action [12], as well as significant differences in the efficacy of individual compounds in the treatment of different forms of depression [2,59]. Based on the present analysis of transcriptional profiles, it would appear that mianserin would be a highly effective treatment for psychotic depression, imipramine for anxiety-depressive disorders, and tranylcypromine for depression associated with anhedonia. The broad gene expression profile of fluoxetine indicates that it would be a suitable first-line treatment [60].

The prediction of drug properties based on the pattern of gene expression alterations need not exactly correspond to the therapeutic profile. To form multidimensional profile of a drug or novel psychoactive compound the results of molecular analysis should be combined with binding profile and behavioral response [61,62]. The pharmacological mechanisms of action of the tricyclic drug tianeptine, indicated for depression, are not fully understood. The present genomic profiling approach appears to have the potential to identify neuronal targets for drugs with unknown mechanisms of action as well as for experimental compounds [63-65]. Until now, tianeptine has been thought to act by either enhancing serotonin reuptake, modulating glutamatergic transmission and/or counteracting maladaptive stress-induced neuroplasticity [24,66]; however, none of these mechanisms has been fully validated. The present study revealed that the transcriptional effects of tianeptine may result from a blockade of norepinephrine, serotonin and dopamine transporters; in this respect, tianpetine shares some of the dopaminergic and noradrenergic properties with its predecessor amineptine [67]. Supporting the view that tianeptine acts primarily by modulating monoaminergic function are clinical findings that the tianeptine, has moderate addictive potential comparable to diazepam [68] as well as the presently observed pattern of tianeptine-induced expression of activity-dependent genes. Importantly, the lack of tianeptine-binding molecular targets suggests that the drug indirectly influences monoamine levels [69]. The transcriptional profile of tianeptine is not necessarily in conflict with the previously proposed mechanisms of its action as positive effects of tianeptine on both glutamatergic transmission and neuroplasticity might be indirect. However, our results suggest a change in tianeptine status from a drug acting through unknown mechanisms to an antidepressant with remarkable ability to modulate all three monoamine systems. Compounds with such activity profile have been recently proposed as likely to form the basis for the development of the next generation of antidepressant drugs [70].

## Conclusions

Psychotropic drugs conventionally classified as antidepressants, antipsychotics, anxiolytics, psychostimulants and opioids regulate expression of three major gene expression networks implicated in the control of neuronal signaling, brain metabolism and organization of cell projections. The patterns of drug-induced gene networks revealed here offer new valuable markers of pharmacological activation of diverse neurobiological processes and systems. In particular, the present study provides novel insights into the mechanisms through which tianeptine might exert its antidepressant action.

## Methods

### Animals

Adult male (8 to 10 weeks old) C57BL/6 J inbred mice (Jackson Laboratory, Bar Harbor, ME, USA) were housed 6 to 10 per cage under a 12-h dark/light cycle with free access to food and water. Animals weighing 20 to 30 g were used throughout the experiments. The animal protocols were approved by the local Bioethics Commission at the Institute of Pharmacology PAS.

### Drug treatment

Mice were injected i.p. (vol. 10 ml/kg) with drugs listed in Table 1. Then animals were sacrificed by decapitation 1, 2, 4 or 8 h after a single injection along with the appropriate vehicle and naïve control groups (6 animals per each drug-treated and control group). Risperidone, haloperidol, clozapine and diazepam were suspended in 1% Tween 80 solution (Sigma-Aldrich, St. Louis, MO, USA); other drugs were dissolved in saline. The effective doses of psychotropic drugs were based on the literature, particular attention being payed to their pharmacological effects in C57BL/6 J mice [71-79]. The doses were selected to provide reasonable comparison of drugs effects on the molecular level.

### Tissue collection and RNA isolation

Samples containing the rostral part of the caudate putamen and the nucleus accumbens, referred to hereafter as the striatum, were collected. The dissection procedure was performed as previously described [80]. In addition, tissue samples containing frontal cortex, amygdalae and hippocampus were frozen in order to allow future experiments. Tissue samples were placed in RNAlater reagent (Qiagen Inc., Valencia, CA, USA) and preserved at −70°C. Samples were homogenized in 1 ml Trizol reagent (Invitrogen, Carlsbad, CA, USA). RNA was isolated following the manufacturer’s protocol and further purified using the RNeasy Mini Kit (Qiagen Inc.). The total RNA concentration was measured using a ND-1000 Spectrometer (NanoDrop Technologies Inc., Montchanin, DE, USA). RNA quality was determined using an Agilent Bioanalyzer 2100 (Agilent, Palo Alto, CA, USA).

### Microarray hybridization

A starting amount of 200 ng high-quality total RNA (pooled 1:1 from two animals) was used to generate cDNA and cRNA with the Illumina TotalPrep RNA Amplification Kit (Illumina Inc., San Diego, CA, USA). The obtained cDNA served as a template for in vitro transcription with T7 RNA polymerase and biotin UTP to generate multiple copies of biotinylated cRNA. Each cRNA sample (1.5 μg) was hybridized overnight to MouseWG-6 BeadChip array (Illumina); subsequently, chips were washed, dried and scanned with the BeadArray Reader (Illumina). Raw microarray data were generated using BeadStudio v3.0 (Illumina). A total of 108 Illumina MouseWG-6 v1.1 and 216 Illumina MouseWG-6 v2 microarrays were used (three independent arrays per group). Samples from 2 mice were pooled per microarray, 3 biological replicates were used per time-point and 12 arrays per each drug. To provide an overall appropriately balanced dataset, treatment group samples were distributed between array plates and hybridization batches.

### Microarray data analysis

Analysis and quality control of 324 microarrays were performed using BeadArray R package v1.10.0. After background subtraction, data was normalized using quantile normalization and then log2-transformed. Results were standardized to reduce the effect of hybridization batches using z-score transformation. All the experiments were planned and performed to allow direct comparison of a relatively large number of psychoactive drugs. Gene cross-annotation between the two versions of Illumina microarrays was performed automatically.

All statistical analyses were performed in R software version 2.11.1 [81]. There were no significant differences in mRNA abundance levels between the batches of vehicle-treated animals (saline as well as Tween 80) after correction for multiple testing. Therefore, for drug comparison all control groups were combined together. Two-way ANOVA with fixed effects for drug factor (df = 19), time factor (df = 3) and interaction (df = 57) was followed by appropriate correction for multiple testing (using the Bonferroni or Benjamini-Hochberg procedures).

The genes2mind gene selection score (implemented on http://www.genes2mind.org) was computed as follows:

$$\mathrm{score}=\frac{\left(10*\left(‒\mathrm{lo}g_{2}p_{\mathrm{ij}}\right)*\mathrm{lo}g_{2}\left(\mathrm{fol}d_{\mathrm{ij}}+1\right)*\mathrm{foldmea}n_{i}\right)}{\mathrm{folds}d_{i}}$$

The variables described: i - drug, j – time-point, p - P value obtained from Student’s t test, fold - fold of change compared to saline control, foldmean - mean fold change from the four experimental time-points and foldsd - standard deviation of fold values from the four time-points. The data integration system was based on MySQL in the data layer, Java in the logic layer and AJAX (GWT) in the presentation layer. Published databases [18,19,82] were used to check cell-type enrichment, mRNA half-life and to control for over-representation of TFBSs of genes [18,19,82]. The functional annotation analysis tool DAVID 2008 was used to identify over-represented ontologic groups among the gene expression patterns [83]. The list of transcripts represented on the Illumina Mouse WG-6 microarray was used as a background list. Over-represented GO terms were defined as having at least three transcripts and P < 0.05 under Fisher’s exact test. The automated functional profiling of drug-regulated genes was performed using the Pathways-Express online tool with default parameters [84].

### Identification of co-expressed gene networks

Spearman correlations were calculated for all pairs of gene expression profiles. A co-expression tree that grouped transcripts with the most similar expression profiles was built using correlation coefficients and a minimal spanning tree algorithm. Visual representation of the data was obtained using the sfdp algorithm from the graphviz R library. Clusters of co-expressed genes were identified using the single-linkage clustering method. Walk-length on the co-expression tree (number of edges separating corresponding transcripts) was used as the distance metric for clustering. The top 300 drug-regulated transcripts were selected (at genes2mind score > 1.8) for clustering. An arbitrary cutoff value (internode distance = 4) was selected to dissect major drug-inducible gene expression networks.

### Model-based inference of pharmacological mechanisms

The pharmacological mechanisms underlying the observed gene expression alterations were transformed into a linear model. Transcriptional effects were modeled as a product of two factors, as follows:

$$E_{\mathrm{transcript}}=\sum_{\mathrm{mech}}A_{\mathrm{mech}}^{\mathrm{transcript}}\cdot B_{\mathrm{drug}}^{\mathrm{mech}}\;$$

Variable A described the sensitivity of transcript abundance to activation levels of a given pharmacological mechanism. The strength of drug-target interaction was represented by the binding parameter B. Its values were based on binding constants found in the PDSP Ki database [25]. The binding matrix contained data on 14 drugs that act through at least one of the 13 pharmacological targets. Together with experimental expression levels, the binding data allowed for the estimation of sensitivity parameters A through a least squares fit. The theoretical model was used to infer the possible mechanisms of tianeptine action. The response matrix A was reduced by finding the 50 most sensitive transcripts for each tested pharmacological mechanism. After removal of duplicates, 350 transcripts were selected for further analysis and their responses to tianeptine were represented by expression vector E'. Together with reduced response matrix A', E' was used in a least squares fit to theoretically predicted tianeptine-induced activation of pharmacological targets. The accuracy of the model was tested by prediction of tranylcypromine mechanism of action (Additional file 9).

### *In situ* hybridization

The frozen brains were cut into 12 μm-thick coronal sections on a cryostat microtome CM 3050S (Leica Microsystems, Germany), and the sections were thaw-mounted on gelatin-chrome alum-coated slides and processed for in situ hybridization. The hybridization procedure was performed as previously described [85]. Briefly, the sections were fixed with 4% paraformaldehyde, washed in PBS and acetylated by incubation with 0.25% acetic anhydrite (in 0.1 M triethanolamine and 0.9% sodium chloride). The sections were dehydrated using increasing concentrations of ethanol (70 to 100%), treated with chloroform for 5 minutes and rehydrated with decreasing concentrations of ethanol. The sections were hybridized for 15 h at 37°C with oligonucleotide probes complementary to *Arc* and *Egr1* cDNA. The probes were labeled with 35S-dATP by the 3'-tailing reaction using terminal transferase (MBI Fermentas, Vilnius, Lithuania). After hybridization, the slices were washed three times for 20 minutes with 1 × SSC/50% formamide at 40°C and twice for 50 minutes with 1 × SSC at room temperature. Then, the slices were dried and exposed to phosphorimager plates (Fujifilm, Tokyo, Japan) for 5 days. The hybridization signal was digitized using a Fujifilm BAS-5000 phosphorimager and Image Reader software (Fujifilm).

### Conditioned place preference

CPP tests were performed using an unbiased procedure in a three-arm apparatus. The experiment consisted of the following phases separated by 24 h: pre-conditioning test (day 0), conditioning with a tianeptine dose of 20 mg/kg (days 1, 3, 5), conditioning with saline (days 2, 4, 6) and post-conditioning test (day 7). The CPP score was defined as the time spent in the drug-paired compartment on day 7 minus the time spent in the same compartment in the preconditioning phase on day 0. The scores were expressed as means with the standard error of the mean.

### URLs

Bioinformatic platforms: genes2mind, http://genes2mind.org; cREMaG database, http://cremag.org; PDSP Ki database, http://pdsp.med.unc.edu.

### Accession codes

Microarray data are available in the NCBI Gene Expression Omnibus (GEO) under accession numbers GEO: GSE15774, GSE48951 and GSE48954.

## Abbreviations

PCA: Principal component analysis; SRE: Serum-response element; CRE: cAMP response element; GRE: Glucocorticoid-response element; GO: Gene ontology; TFBS: Transcription factor binding site; CPP: Conditioned place preference.

## Competing interests

The authors declare that they have no competing financial interests.

## Authors’ contributions

MK designed the study, interpreted the results and drafted the manuscript. MP, WM and JD performed statistical analyses and developed bioinformatics tools. KS conducted behavioral testing. BZ was responsible for *in situ* hybridization experiments. RP coordinated the study and reviewed the manuscript. All authors read and approved the final manuscript.

## Supplementary Material

Additional file 1 **A table listing the results of the two-way ANOVA for drug treatment factor (followed by Bonferroni or FDR corrections for multiple tests).** For drug-regulated transcripts genes2mind scores and gene network associations are provided. The second sheet contains lists of the genes from the three major gene expression networks α, β and γ. Click here for file

Additional file 2 **ANOVA results of gene expression profiling of drug effects in mouse striatum.** A figure presenting the relationship between the number of true positive results and the proportion of false positives for drug factor in ANOVA. Click here for file

Additional file 3 **A table listing the comparison of transcriptional effects of the tested psychotropic drugs.** The table contains the number of regulated transcripts (genes2mind score >10) at each time-point of the experiment (obtained using the genes2mind gene selection module). Click here for file

Additional file 4 **A figure showing a minimal spanning tree of the whole-transcriptome, based on correlation of gene expression profiles.** Each node represents one transcript (an example branch with 4 transcripts was presented on the right). The internode distance is proportional to the Spearman correlation of the expression levels of two transcripts. The top 300 drug-responsive genes are depicted by red color (defined by genes2mind score using the four time-points). Click here for file

Additional file 5 **A figure showing hierarchical clustering of drug-induced gene expression alterations in the mouse striatum.** Microarray results are shown as a heat map and include 872 transcripts with a significance (FDR < 5%) obtained from two-way analysis of variance of the drug factor. Colored rectangles represent transcript abundance 1, 2, 4 and 8 h after injection of the drug indicated above. The intensity of the color is proportional to the standardized values from each microarray. Drug-responsive gene networks were denoted on the right. Click here for file

Additional file 6 **A table listing the complete results of the GO analysis presented in the manuscript.** The analyses were performed on lists of genes that correspond to networks α, β and γ (results are presented in separate sheets). The analyzed genes are listed in Additional file 1. Click here for file

Additional file 7 **A table listing the complete results of the canonical pathways analysis presented in the manuscript.** The analyses were performed on extended (FDR < 5%) lists of genes that correspond to networks α, β and γ (results are presented in separate sheets). The analyzed genes are listed in Additional file 1. Click here for file

Additional file 8 **A figure showing examples of canonical biological pathways regulated by psychotropic drugs.** The analyses were performed on extended (FDR < 5%) lists of genes that correspond to networks patterns α, β and γ. The pathways were created based on KEGG database using the Pathways-Express online tool. Drug-responsive genes were indicated using yellow color. Click here for file

Additional file 9 **The mechanisms of tranylcypromine action predicted from expression profiles of the transcripts most sensitive to the analyzed pharmacological mechanisms (for details please see **Methods** section).** Click here for file
