# Supplementary figures and images for: Novel drug-regulated transcriptional networks in brain reveal pharmacological properties of psychotropic drugs
Source: BMC Genomics. 2013 Sep 8;14:606. doi: 10.1186/1471-2164-14-606 (PMC3844597; doi:10.1186/1471-2164-14-606)

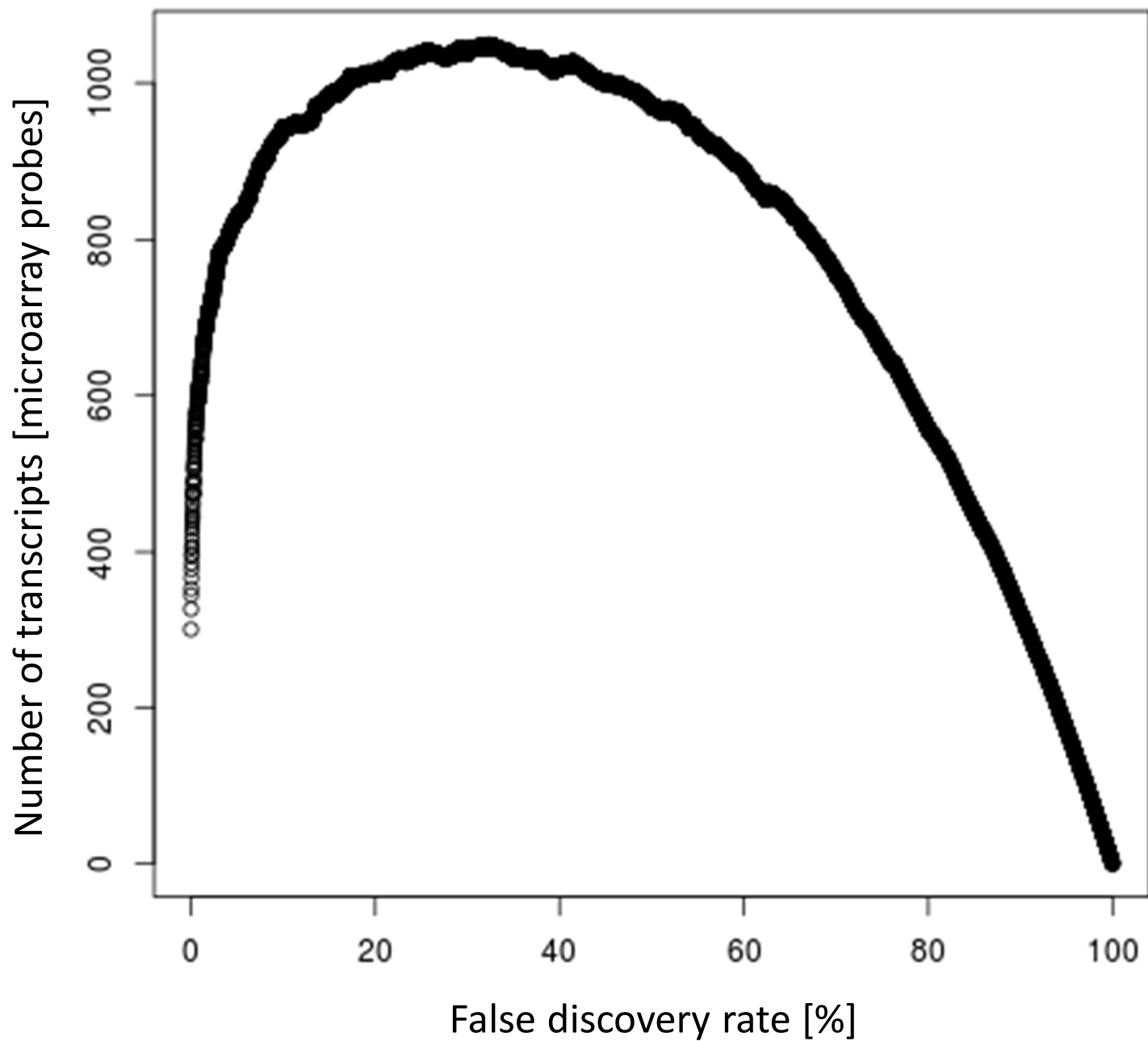

Supplement: Additional file 2 — ANOVA results of gene expression profiling of drug effects in mouse striatum. A figure presenting the relationship between the number of true positive results and the proportion of false positives for drug factor in ANOVA. [file 1471-2164-14-606-S2.pdf]

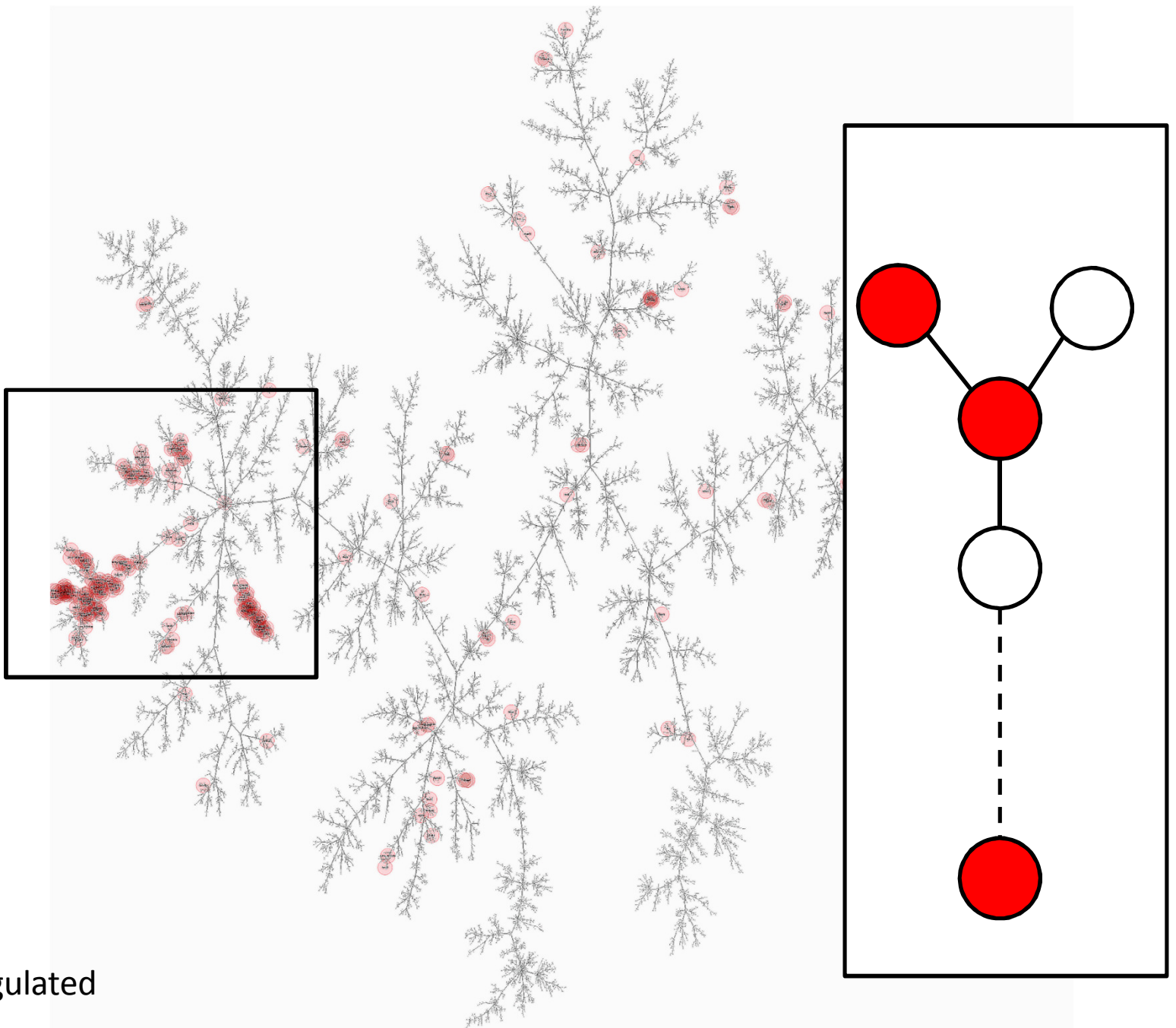

300 drug-regulated  
transcripts

Supplement: Additional file 4 — A figure showing a minimal spanning tree of the whole-transcriptome, based on correlation of gene expression profiles. Each node represents one transcript (an example branch with 4 transcripts was presented on the right). The internode distance is proportional to the Spearman correlation of the expression levels of two transcripts. The top 300 drug-responsive genes are depicted by red color (defined by genes2mind score using the four time-points). [file 1471-2164-14-606-S4.pdf]

**Drug-responsive genes selected at <5% FDR.**

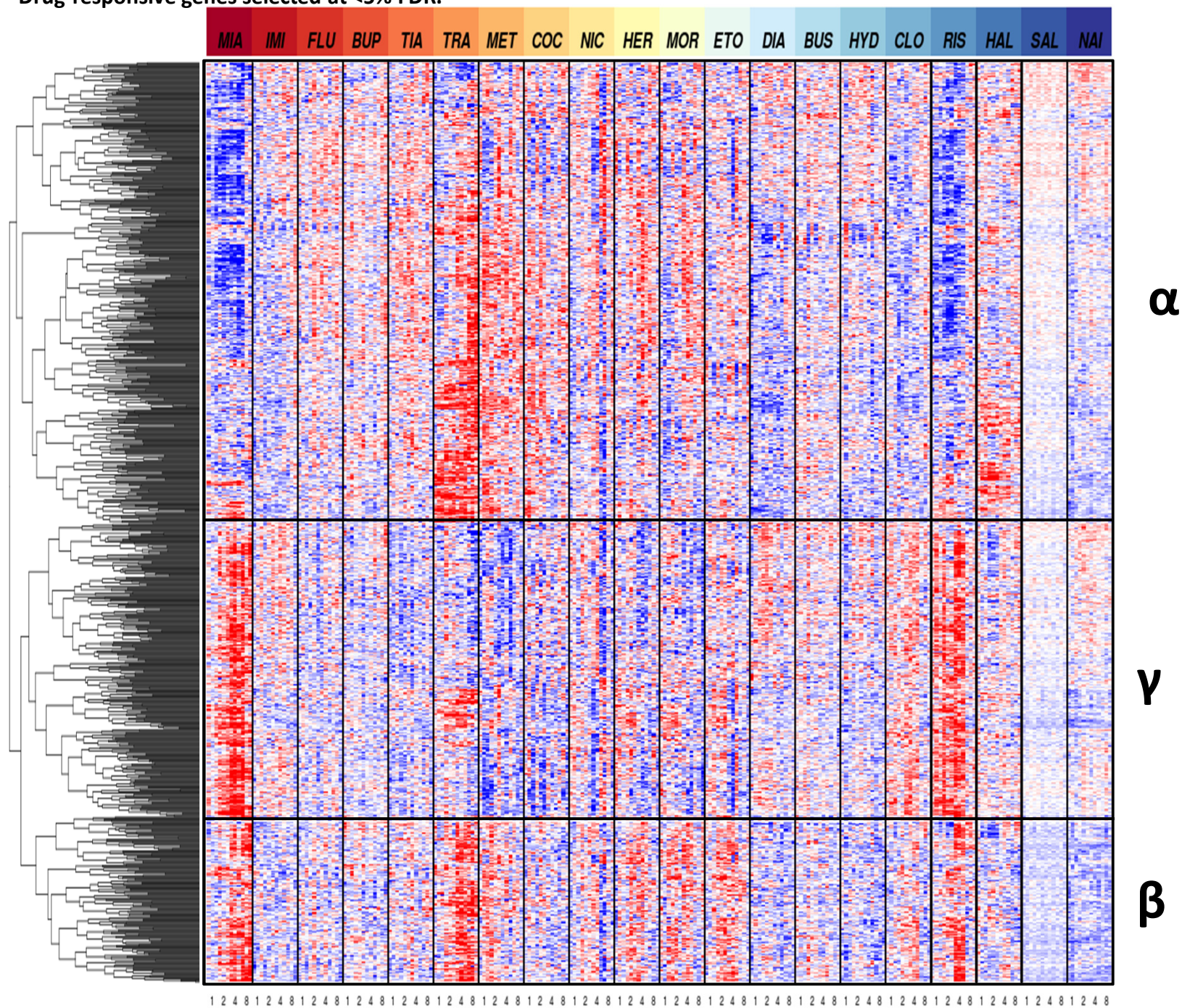

Supplement: Additional file 5 — A figure showing hierarchical clustering of drug-induced gene expression alterations in the mouse striatum. Microarray results are shown as a heat map and include 872 transcripts with a significance (FDR < 5%) obtained from two-way analysis of variance of the drug factor. Colored rectangles represent transcript abundance 1, 2, 4 and 8 h after injection of the drug indicated above. The intensity of the color is proportional to the standardized values from each microarray. Drug-responsive gene networks were denoted on the right. [file 1471-2164-14-606-S5.pdf]

# TRANLYCYPROMINE

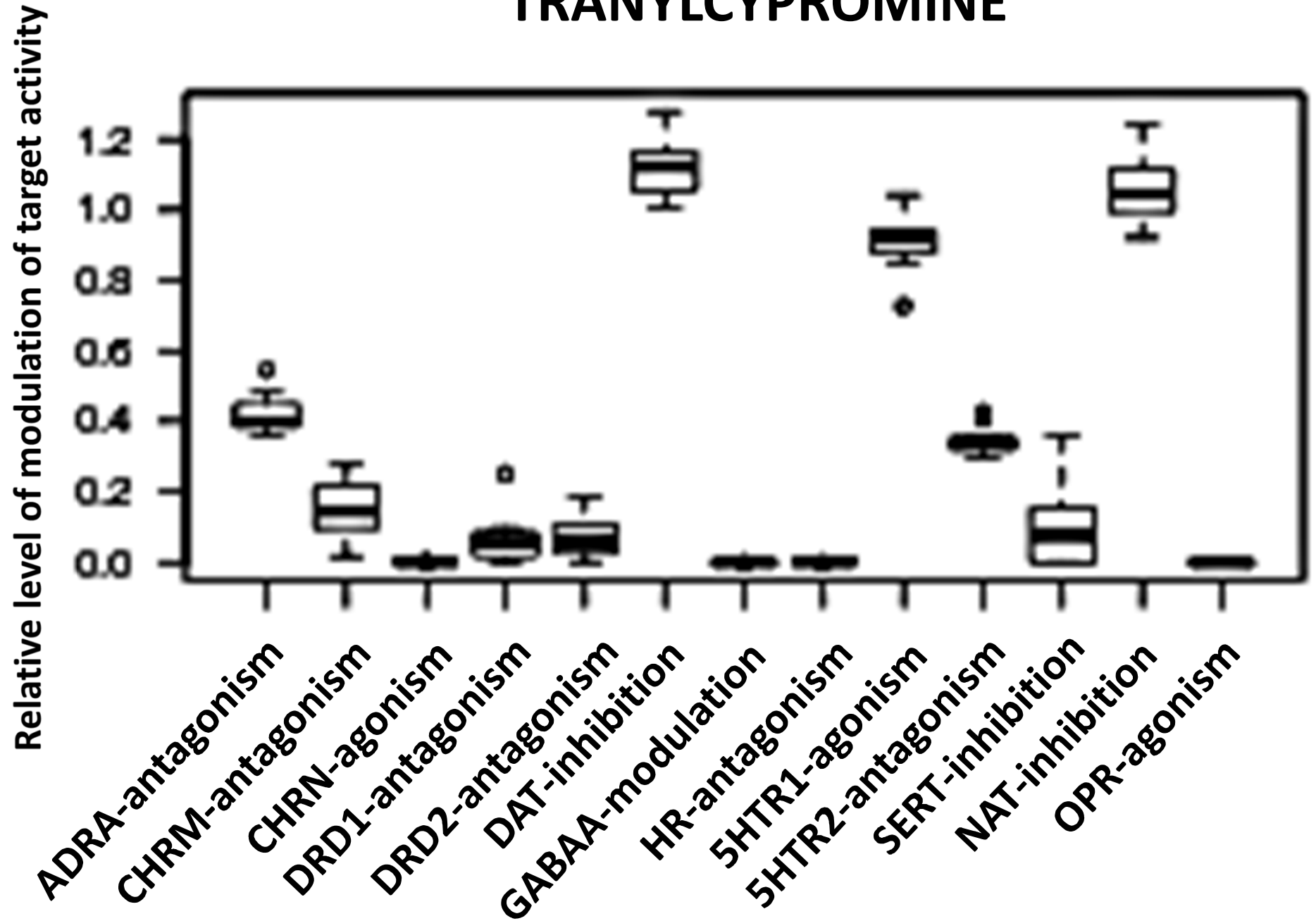

Supplement: Additional file 9 — The mechanisms of tranylcypromine action predicted from expression profiles of the transcripts most sensitive to the analyzed pharmacological mechanisms (for details please see Methods section). [file 1471-2164-14-606-S9.pdf]
