# Supplementary material for: Novel drug-regulated transcriptional networks in brain reveal pharmacological properties of psychotropic drugs
Source: BMC Genomics. 2013 Sep 8;14:606. doi: 10.1186/1471-2164-14-606 (PMC3844597; doi:10.1186/1471-2164-14-606)
Supplement: Additional file 8 — A figure showing examples of canonical biological pathways regulated by psychotropic drugs. The analyses were performed on extended (FDR < 5%) lists of genes that correspond to networks patterns α, β and γ. The pathways were created based on KEGG database using the Pathways-Express online tool. Drug-responsive genes were indicated using yellow color. [file 1471-2164-14-606-S8.pdf]

# α network

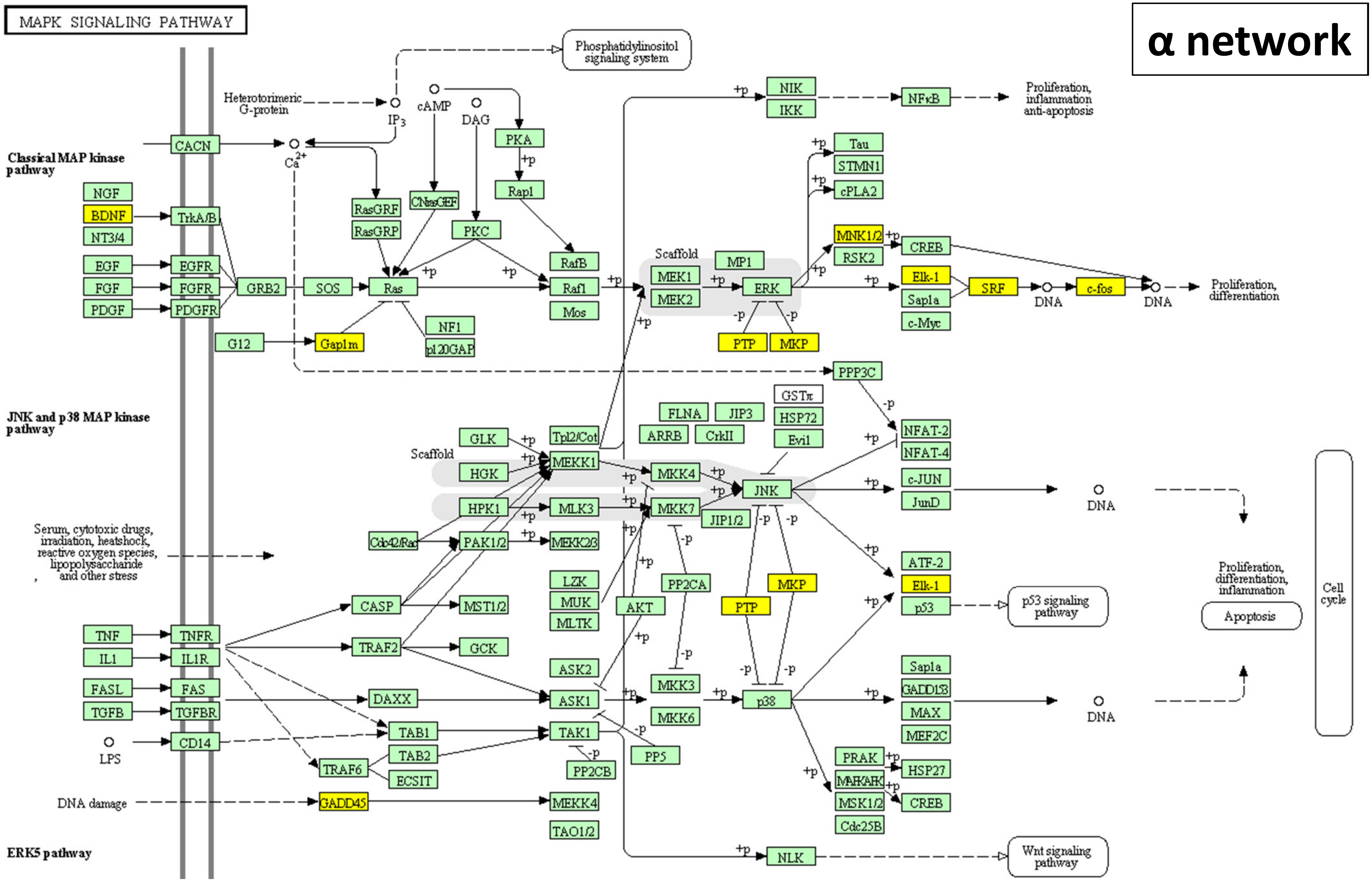
MAPK SIGNALING PATHWAY
Phosphatidylinositol signaling system
Heterotorimeric G-protein
IP3
cAMP
DAG
Classical MAP kinase pathway
CACN
Ca2+
PKA
Rap1
NIK
IKK
NFκB
Proliferation, inflammation anti-apoptosis
Tau
STMN1
cPLA2
MNK1/2
RSK2
CREB
NGF
BDNF
NT3/4
TrkA/B
RasGRF
RasGRP
CNrasGEF
PKC
RafB
Scaffold
MEK1
MEK2
MP1
ERK
Elk-1
Sap1a
c-Myc
SRF
DNA
c-fos
DNA
Proliferation, differentiation
EGF
FGF
PDGF
EGFR
FGFR
PDGFR
GRB2
SOS
Ras
Raf1
Mos
NF1
p120GAP
G12
Gap1m
PTP
MKP
PPP3C
JNK and p38 MAP kinase pathway
GSTπ
HSP72
Evi1
FLNA
JIP3
ARRB
CrkII
NFAT-2
NFAT-4
GLK
Tpl2/Cot
Scaffold
HGK
MEKK1
MKK4
JNK
c-JUN
JunD
DNA
HPK1
MLK3
MKK7
JIP1/2
Serum, cytotoxic drugs, irradiation, heatshock, reactive oxygen species, lipopolysaccharide and other stress
Cdc42/Rac
PAK1/2
MEKK2/3
LZK
MUK
MLTK
PP2CA
MKP
ATF-2
Elk-1
p53
p53 signaling pathway
Proliferation, differentiation, inflammation
Apoptosis
Cell cycle
CASP
MST1/2
AKT
PTP
TNF
IL1
TNFR
IL1R
TRAF2
GCK
ASK2
FASL
TGFB
FAS
TGFBR
DAXX
ASK1
MKK3
MKK6
p38
Sap1a
GADD153
MAX
MEF2C
DNA
LPS
CD14
TAB1
TAB2
TRAF6
ECSIT
TAK1
PP2CB
PP5
PRAK
MAPKAPK
MSK1/2
Cdc25B
HSP27
CREB
DNA damage
GADD45
MEKK4
TAO1/2
ERK5 pathway
NLK
Wnt signaling pathway

# α network

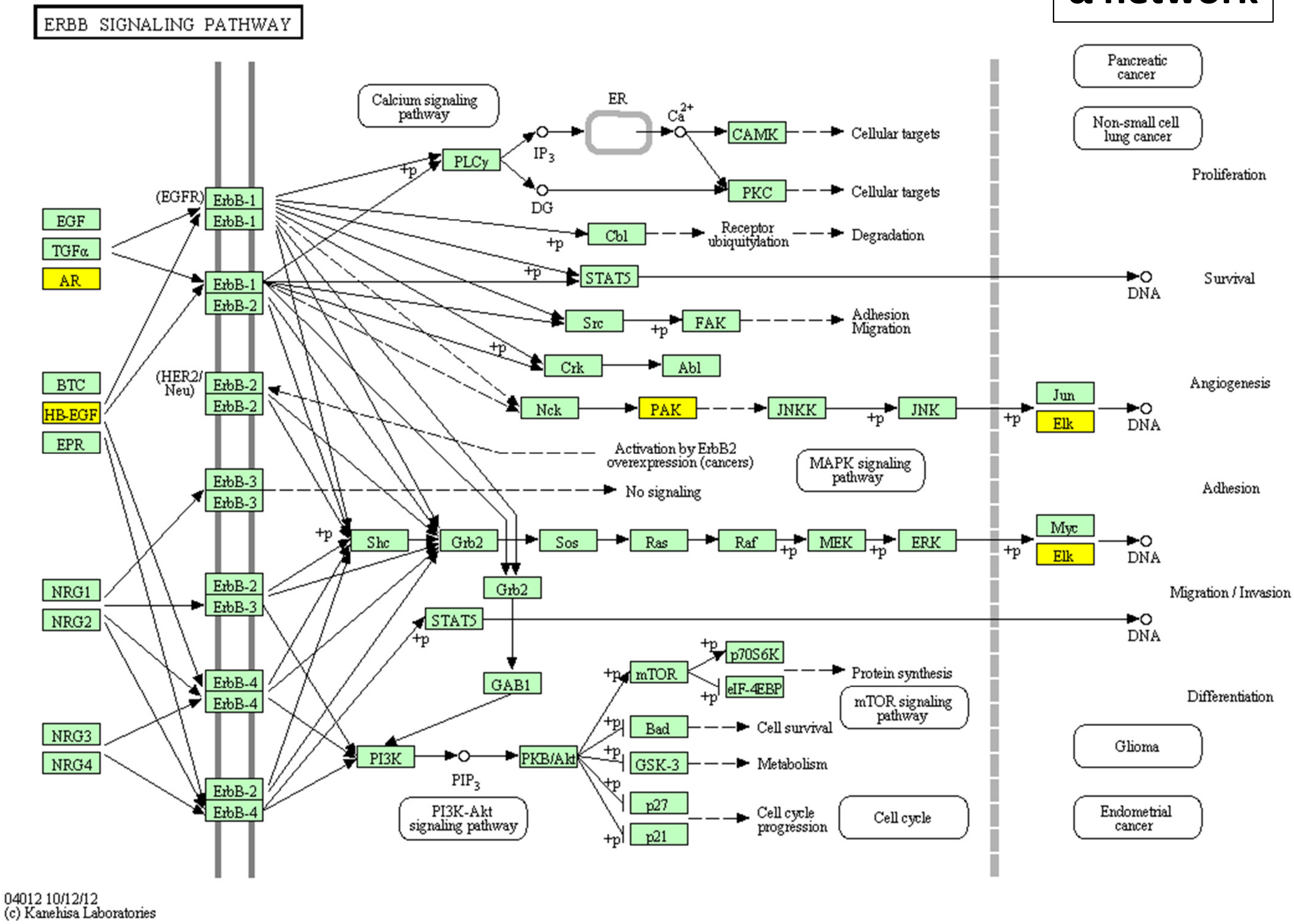
ERBB SIGNALING PATHWAY
EGF
TGFα
AR
BTC
HB-EGF
EPR
NRG1
NRG2
NRG3
NRG4
(EGFR)
ErbB-1
ErbB-1
ErbB-1
ErbB-2
(HER2/Neu)
ErbB-2
ErbB-2
ErbB-3
ErbB-3
ErbB-2
ErbB-3
ErbB-4
ErbB-4
ErbB-2
ErbB-4
Calcium signaling pathway
PLCγ
IP3
DG
ER
Ca2+
CAMK
PKC
Cellular targets
Cellular targets
Cbl
Receptor ubiquitylation
Degradation
STAT5
DNA
Src
FAK
Adhesion Migration
Crk
Abl
Nck
PAK
JNKK
JNK
Jun
Elk
DNA
Activation by ErbB2 overexpression (cancers)
MAPK signaling pathway
No signaling
Shc
Grb2
Sos
Ras
Raf
MEK
ERK
Myc
Elk
DNA
Grb2
STAT5
DNA
GAB1
PI3K
PIP3
PKB/Akt
PI3K-Akt signaling pathway
mTOR
p70S6K
eIF-4EBP
Protein synthesis
mTOR signaling pathway
Bad
Cell survival
GSK-3
Metabolism
p27
p21
Cell cycle progression
Cell cycle
+p
Pancreatic cancer
Non-small cell lung cancer
Proliferation
Survival
Angiogenesis
Adhesion
Migration / Invasion
Differentiation
Glioma
Endometrial cancer
04012 10/12/12
(c) Kanehisa Laboratories

# β network

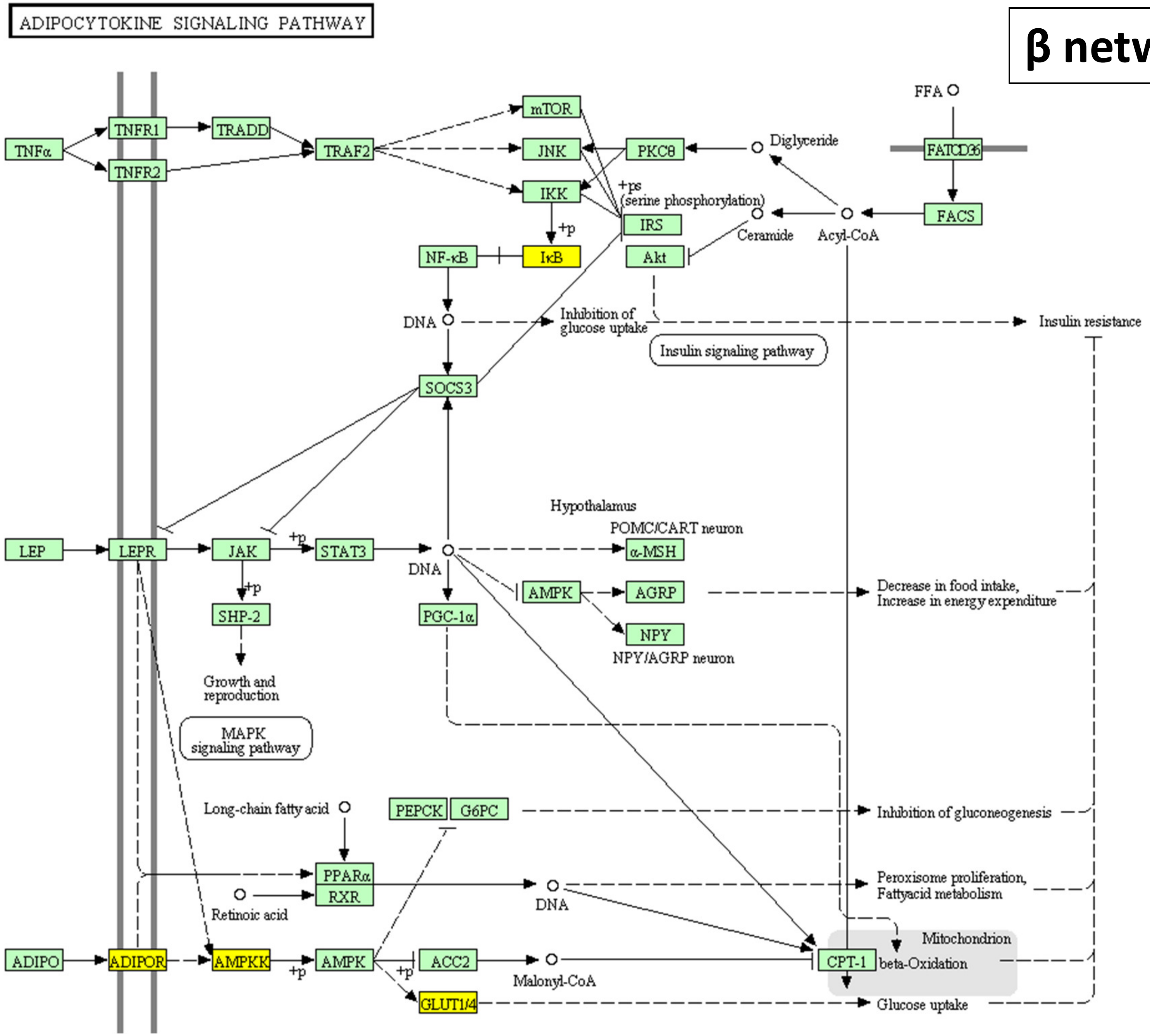
ADIPOCYTOKINE SIGNALING PATHWAY
TNFα
TNFR1
TNFR2
TRADD
TRAF2
mTOR
JNK
IKK
PKCθ
Diglyceride
FFA
FAT/CD36
FACS
Acyl-CoA
Ceramide
+ps (serine phosphorylation)
IRS
Akt
+p
NF-κB
IκB
DNA
Inhibition of glucose uptake
Insulin signaling pathway
Insulin resistance
SOCS3
Hypothalamus
POMC/CART neuron
α-MSH
LEP
LEPR
JAK
+p
STAT3
DNA
AMPK
AGRP
NPY
NPY/AGRP neuron
Decrease in food intake, Increase in energy expenditure
+p
SHP-2
PGC-1α
Growth and reproduction
MAPK signaling pathway
Long-chain fatty acid
PEPCK
G6PC
Inhibition of gluconeogenesis
PPARα
RXR
Retinoic acid
DNA
Peroxisome proliferation, Fattyacid metabolism
Mitochondrion
ADIPO
ADIPOR
AMPKK
+p
AMPK
+p
ACC2
Malonyl-CoA
CPT-1
beta-Oxidation
GLUT1/4
Glucose uptake

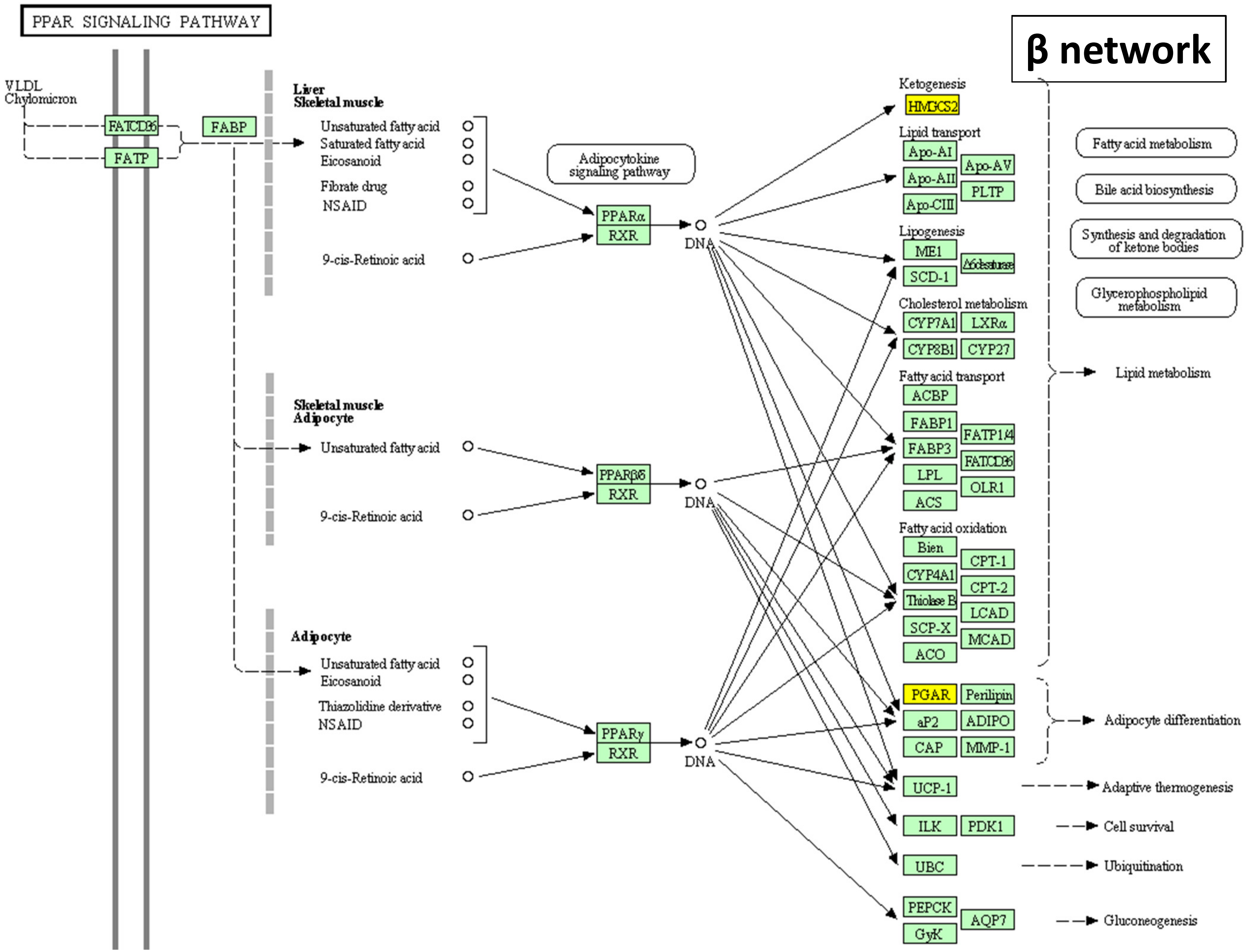
β network
PPAR SIGNALING PATHWAY
VLDL
Chylomicron
FATCD36
FATP
FABP
Liver
Skeletal muscle
Unsaturated fatty acid
Saturated fatty acid
Eicosanoid
Fibrate drug
NSAID
9-cis-Retinoic acid
Adipocytokine signaling pathway
PPARα
RXR
DNA
Skeletal muscle
Adipocyte
Unsaturated fatty acid
9-cis-Retinoic acid
PPARβ/δ
RXR
DNA
Adipocyte
Unsaturated fatty acid
Eicosanoid
Thiazolidine derivative
NSAID
9-cis-Retinoic acid
PPARγ
RXR
DNA
Ketogenesis
HMGCS2
Lipid transport
Apo-AI
Apo-AII
Apo-CIII
Apo-AV
PLTP
Lipogenesis
ME1
SCD-1
Δ6desaturase
Cholesterol metabolism
CYP7A1
CYP8B1
LXRα
CYP27
Fatty acid transport
ACBP
FABP1
FABP3
LPL
ACS
FATP1/4
FATCD36
OLR1
Fatty acid oxidation
Bien
CYP4A1
Thiolase B
SCP-X
ACO
CPT-1
CPT-2
LCAD
MCAD
PGAR
aP2
CAP
Perilipin
ADIPO
MMP-1
UCP-1
ILK
PDK1
UBC
PEPCK
GyK
AQP7
Fatty acid metabolism
Bile acid biosynthesis
Synthesis and degradation of ketone bodies
Glycerophospholipid metabolism
Lipid metabolism
Adipocyte differentiation
Adaptive thermogenesis
Cell survival
Ubiquitination
Gluconeogenesis

# γ network

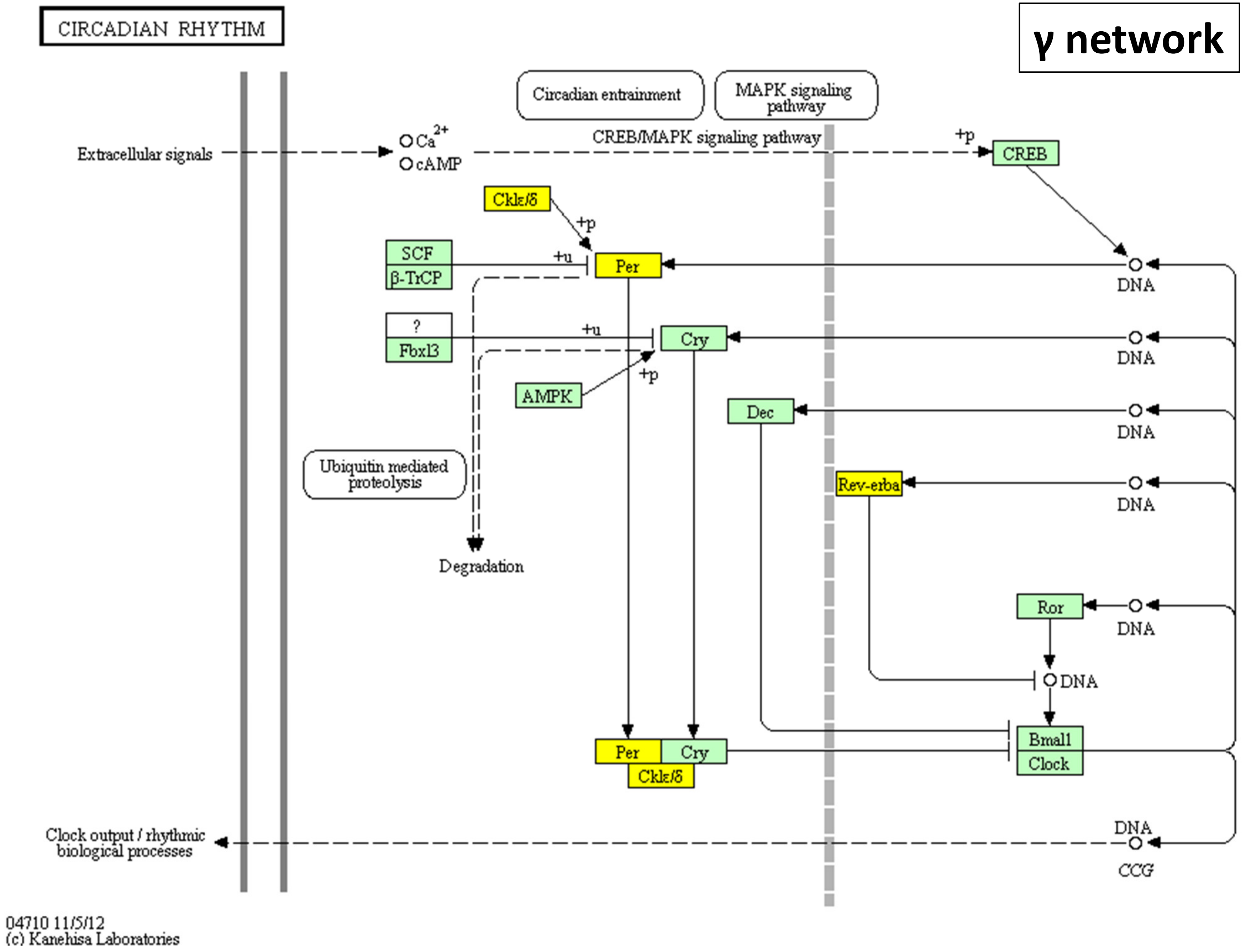
CIRCADIAN RHYTHM
Circadian entrainment
MAPK signaling pathway
Extracellular signals
Ca2+
cAMP
CREB/MAPK signaling pathway
+p
CREB
Cklε/δ
+p
SCF
β-TrCP
+u
Per
DNA
?
Fbxl3
+u
Cry
DNA
AMPK
+p
Dec
DNA
Ubiquitin mediated proteolysis
Rev-erba
DNA
Degradation
Ror
DNA
DNA
Bmal1
Clock
Per
Cry
Cklε/δ
Clock output / rhythmic biological processes
DNA
CCG
04710 11/5/12
(c) Kanehisa Laboratories

γ network

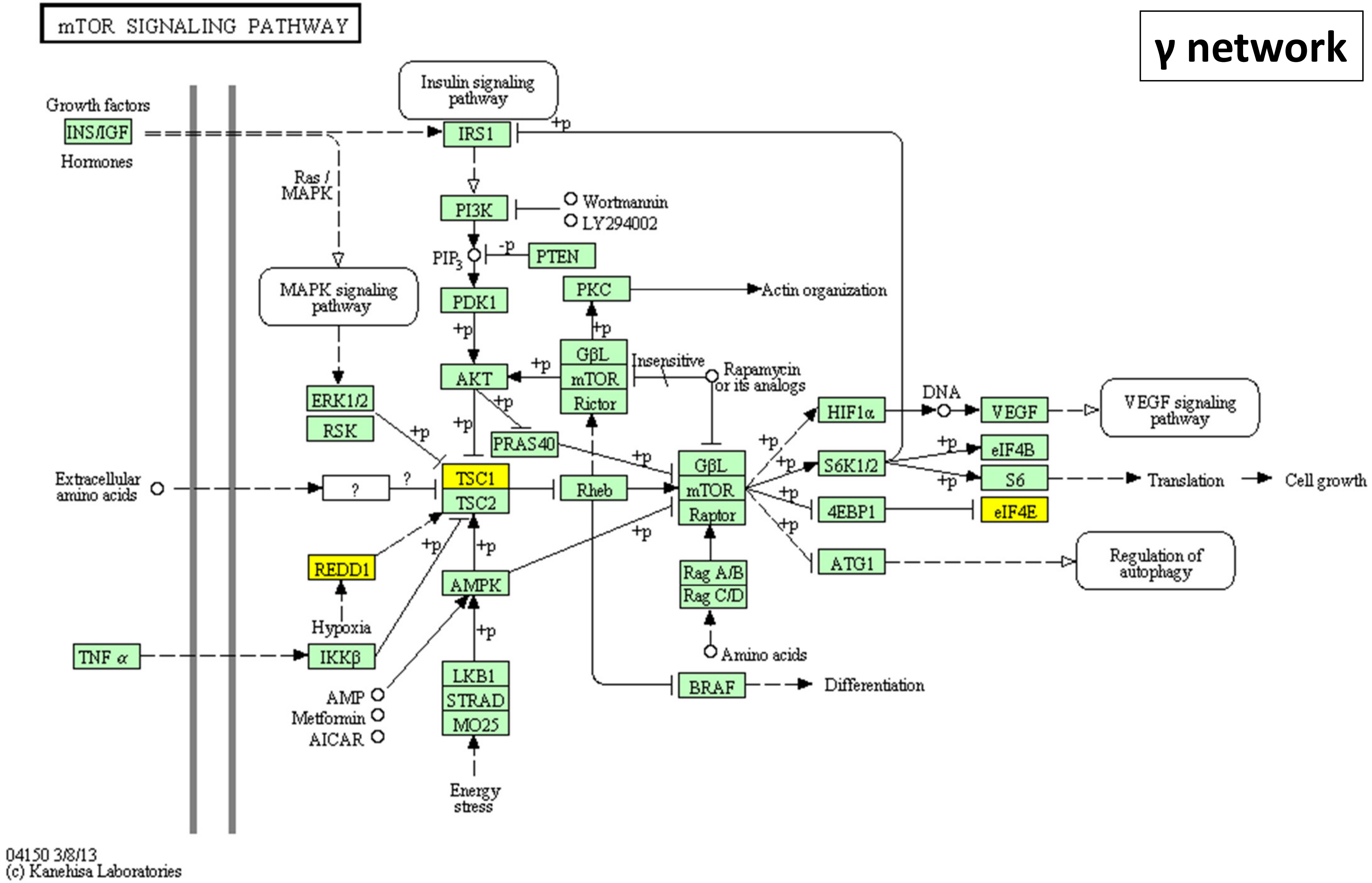
mTOR SIGNALING PATHWAY
Growth factors
INS/IGF
Hormones
Insulin signaling pathway
IRS1
+p
Ras / MAPK
PI3K
Wortmannin
LY294002
PIP3
-p
PTEN
PKC
Actin organization
MAPK signaling pathway
PDK1
+p
GβL
mTOR
Rictor
AKT
Insensitive
Rapamycin or its analogs
ERK1/2
RSK
PRAS40
HIF1α
DNA
VEGF
VEGF signaling pathway
Extracellular amino acids
?
TSC1
TSC2
Rheb
GβL
mTOR
Raptor
S6K1/2
eIF4B
S6
Translation
Cell growth
4EBP1
eIF4E
ATG1
Regulation of autophagy
REDD1
AMPK
Rag A/B
Rag C/D
Hypoxia
TNF α
IKKβ
Amino acids
LKB1
STRAD
MO25
BRAF
Differentiation
AMP
Metformin
AICAR
Energy stress
04150 3/8/13
(c) Kanehisa Laboratories
